# A Computationally Efficient Reconstruction Algorithm for Circular Cone-Beam Computed Tomography Using Shallow Neural Networks

**DOI:** 10.3390/jimaging6120135

**Published:** 2020-12-08

**Authors:** Marinus J. Lagerwerf, Daniël M. Pelt, Willem Jan Palenstijn, Kees Joost Batenburg

**Affiliations:** 1 Centrum Wiskunde & Informatica, Science Park 123, 1098 XG Amsterdam, The Netherlands; d.m.pelt@liacs.leidenuniv.nl (D.M.P.); wjp@cwi.nl (W.J.P.); k.j.batenburg@cwi.nl (K.J.B.); 2 Leiden Institute of Advanced Computer Science, Universiteit Leiden, 2333 CA Leiden, The Netherlands

**Keywords:** tomography, circular cone-beam CT, machine learning, neural network, multilayer perceptron, Feldkamp–Davis–Kress (FDK), reconstruction algorithm

## Abstract

Circular cone-beam (CCB) Computed Tomography (CT) has become an integral part of industrial quality control, materials science and medical imaging. The need to acquire and process each scan in a short time naturally leads to trade-offs between speed and reconstruction quality, creating a need for fast reconstruction algorithms capable of creating accurate reconstructions from limited data. In this paper, we introduce the Neural Network Feldkamp–Davis–Kress (NN-FDK) algorithm. This algorithm adds a machine learning component to the FDK algorithm to improve its reconstruction accuracy while maintaining its computational efficiency. Moreover, the NN-FDK algorithm is designed such that it has low training data requirements and is fast to train. This ensures that the proposed algorithm can be used to improve image quality in high-throughput CT scanning settings, where FDK is currently used to keep pace with the acquisition speed using readily available computational resources. We compare the NN-FDK algorithm to two standard CT reconstruction algorithms and to two popular deep neural networks trained to remove reconstruction artifacts from the 2D slices of an FDK reconstruction. We show that the NN-FDK reconstruction algorithm is substantially faster in computing a reconstruction than all the tested alternative methods except for the standard FDK algorithm and we show it can compute accurate CCB CT reconstructions in cases of high noise, a low number of projection angles or large cone angles. Moreover, we show that the training time of an NN-FDK network is orders of magnitude lower than the considered deep neural networks, with only a slight reduction in reconstruction accuracy.

## 1. Introduction

Circular cone-beam (CCB) Computed Tomography (CT) has become an integral part of non-destructive imaging in a broad spectrum of applications, such as industrial quality control [1], materials sciences [2,3] and medical imaging [4,5]. Especially in industrial and medical applications, the scanning time, reconstruction time and the scanning dose are limited resources. Such limitations lead to trade-offs between computation time and scanning time—i.e., number of projections, noise level—on the one hand, and reconstruction accuracy on the other hand. Additionally, CT reconstruction has become a *big data* problem due to the development of readily available high-resolution CT-scanners [6,7,8]. This stresses the need for computationally efficient reconstruction methods that are applicable to a broad spectrum of high-resolution problems and produce accurate results from data with high noise levels, low numbers of projection angles or large cone angles.

In practice, if computational efficiency is a constraint and especially for high-resolution problems, *direct methods* (e.g., the filtered backprojection (FBP) algorithm [9], the Feldkamp–Davis–Kress (FDK) algorithm [10] and the Katsevich algorithm [11]) are still the common choice of reconstruction method [12]. While *iterative methods* have been shown to be more accurate for noisy and limited data problems [13,14,15,16,17,18], they have a significantly higher computational cost. Consequently, there have been efforts to improve the accuracy of direct methods by computing data-specific or scanner-specific filters [19,20,21,22,23]. Although these strategies do improve the reconstruction accuracy, they also add significant computational effort or are specific to one modality, e.g., tomosynthesis [24].

An emerging approach for improving direct methods is to use *machine learning* to remove artifacts from the reconstructions. The idea is to use high-quality reconstructions to train a neural network that removes artifacts from low-quality reconstructions using a supervised learning approach. This *post-processing* approach has shown promising results for computed tomography using deep neural networks (DNNs) [25,26,27]. Deep neural network structures contain a large number of layers, leading to millions of trainable parameters and, therefore, require a large amount of training data [28]. This is problematic in CT imaging, since there is often a limited amount of training data available, e.g., due to scanning time, dose, and business-related concerns. Moreover, for the available data, there are often no reference datasets or annotations available [29]. The large amount of training data and large number of parameters also lead to long training times. While for standard 2D networks, the training time ranges between a couple of hours and a couple of days (see Section 5.1.2), for 3D networks the training time becomes prohibitively long [30] (i.e., weeks). Therefore, to apply post-processing to 3D problems, the reconstruction volume can be considered as a stack of 2D problems [26,31] for which one 2D network is trained and then applied in a *slice-by-slice* fashion to the 3D volume. Although this strategy reduces the training time and the training data constraints, applying a 2D network to all slices can still be computationally intensive due to the number of slices in the 3D volume. A more in-depth discussion on current developments related to machine learning methods in CT imaging is given in Section 2.

In this work, we propose the Neural Network FDK (NN-FDK) reconstruction algorithm. It is a direct reconstruction method that is designed to produce accurate results from noisy data, data with a low number of projection angles, or a large cone angle, but still maintains a similar computational efficiency and scalability as the standard FDK algorithm. Moreover, the algorithm has a fast training procedure, and requires a limited amount of training data.

The NN-FDK algorithm is an adaptation of the standard FDK algorithm using a shallow multilayer perceptron network [32] with one fully connected hidden layer, a low number of trainable parameters and low memory constraints. We will show it is possible to interpret the weights of the first layer of the perceptron network as a set of learned filters for the FDK algorithm. We can then use the FDK algorithm to evaluate the network efficiently for all voxels simultaneously to arrive at an accurate reconstruction for the CCB CT problem.

The NN-FDK algorithm is an extension of the method proposed in [33] for the Filtered Backprojection (FBP) algorithm [9]. The derivation of the approach outlined in [33] relies on the shift-invariance property of the FBP algorithm. We will show that, although the FDK algorithm does not have this shift-invariance property, we can derive a similar method for the FDK algorithm. Moreover, the proposed strategy can be extended to any linear filtered backprojection type reconstruction method.

Using both simulated and experimental data, we compare the proposed method with the standard FDK algorithm, SIRT [34] with a nonnegativity constraint (SIRT^+^), which is a commonly used iterative algorithm for CT problems, and two 2D deep neural networks (U-net [31] and MSD-net [26]) trained to remove reconstruction artifacts from slices of standard FDK reconstruction. We show that the NN-FDK algorithm is faster to evaluate than all but the standard FDK algorithm and orders of magnitude faster to train than the considered DNNs, with only a slight reduction in reconstruction accuracy compared to the DNNs.

The paper is structured as follows. In Section 3, we give definitions and introduce our method. In Section 4, we introduce the data and the parameters used for the experiments. The experiments and their results are shown and discussed in Section 5. The paper is summarized and concluded in Section 6.

## 2. Related Work

Using *machine learning* methods is an emerging approach in CT imaging [29]. *Deep learning* methods have shown promising results for many applications within the development of CT reconstruction methods [35]. For the sake of exposition, we split these machine learning approaches into two categories: (i) Improving standard reconstruction methods by replacing components of the reconstruction method with networks specifically trained for the application; and (ii) improving the image quality of reconstructions computed with existing reconstruction methods by training neural networks to perform *post-processing* in order to remove artifacts or reduce noise.

Examples of the first strategy (improving standard reconstruction methods) applied to iterative methods are the learned primal-dual reconstruction algorithm [36,37], variational networks [38,39], plug and play priors [40,41,42], and learned regularizers [43,44]. These methods achieve promising results in reconstruction accuracy and generalizability. However, their high computational cost limits the applicability if high throughput is required. Examples for this strategy applied to direct methods are the NN-FBP method [33], and also the NN-FDK method introduced in this paper. These methods are designed to improve the image quality of direct methods for data with limitations (e.g., data with noise or a low number of projection angles), while maintaining their computational efficiency.

Examples of the second strategy (learned post-processing) have demonstrated substantial improvements in reconstruction quality for CT imaging [25,28,31,35]. This is aided by the fact that the post-processing problem can be viewed as a classic imaging problem—e.g., denoising, segmentation, inpainting, classification— for which many effective machine learning methods have already been developed [45,46,47]. Although the general trend is towards deeper networks to make such networks more expressive [48], this can lead to problems with scalability for large 3D image datasets.

The rise in popularity of machine learning in CT is driven by the increased computational possibilities and although these advances are sufficient to handle most 2D problems, scaling towards 3D problems can be problematic, due to memory constraints. This is illustrated in Section 5.1.1, where we plotted the memory constraints for applying a 2D and 3D U-net and MSD-net in terms of gigabytes (GB) of memory as a function of the size of the image. This shows that, in theory, one could apply a 2D MSD-net to images of 7500×7500 pixels (with a 24GB GPU), but in 3D, this limit lies around 400×400×400 voxels. Considering that CT problems range between 256×256×256 (small image size) up to 4096×4096×4096 images, this gives an indication that scalability can become an issue, especially for 3D problems.

When applying machine learning techniques for improving the reconstruction quality in CT, a balance must be struck between image quality, running time, and memory requirements. Here, we propose a method that achieves relatively high accuracy, while also being computationally efficient and scalable.

## 3. Method

The NN-FDK algorithm is a reconstruction algorithm with a machine learning component, meaning that a number of parameters of the reconstruction algorithm are optimized through *supervised learning* [49]. Similar to the network presented in [33], the *NN-FDK network* is a two layer neural network with a hidden layer and an output layer. We design the network such that it reconstructs one single voxel, but handles all voxels in a similar manner. This means that we only have to train one network for a full reconstruction. We consider the NN-FDK algorithm to have three parts: The *NN-FDK network*, the *NN-FDK reconstruction algorithm* and the *training process*.

We introduce the reconstruction problem, FDK algorithm, a filter approximation method and the definition of a perceptron in Section 3.1. In Section 3.2, we provide the *NN-FDK reconstruction algorithm* and derive from this algorithm the *NN-FDK network*. The input of the network that is needed in the *training process* is a pre-processed version of the input of the reconstruction algorithm. In Section 3.3, we discuss how to compute this pre-processing step for all voxels simultaneously and we introduce the optimization problem and related notation for the training process. Lastly, we summarize and discuss the characteristics of the method in Section 3.4.

### 3.1. Preliminaries

#### 3.1.1. Reconstruction Problem

In this paper, we focus exclusively on the circular cone-beam (CCB) geometry, where the object rotates with respect to a point source and a planar detector, acquiring 2D cone-beam projections. The reconstruction problem for the CCB geometry can be modeled by a system of linear equations

(1) $$\begin{array}{c} Wx=y, \end{array}$$

where $x\in R^{n}$ is the vector describing the reconstruction (i.e., every element coincides with a voxel value), $y\in R^{m}$ is the vector describing the measured projection data, and $W\in R^{m\times n}$ is a discretized version of the *cone-beam transform* or *forward projection*. For the sake of simplicity, we assume that the volume consists of n=N×N×N voxels and the detector consists of N×N pixels. We denote the number of angles by $N_{a}$, so we have $m=N_{a}\times N\times N$.

#### 3.1.2. FDK Algorithm & Filter Approximation

The FDK algorithm, as presented in [10], is a filtered backprojection-type algorithm that solves the CCB reconstruction problem (1) approximately. First, for each projection angle, it applies a *reweighting* step, $r:R^{N_{a}\times N\times N}\to R^{N_{a}\times N\times N}$, that adapts the cone-beam data such that it approximately behaves as fan-beam data. Second, it applies a *filtering* step, that convolves the data with a one-dimensional *filter* h in a line-by-line fashion, ${\left(-*-\right)}_{1D}:R^{2N}\times R^{N_{a}\times N\times N}\to R^{N_{a}\times N\times N}$. Last, it applies a *backprojection* step. This step transforms the filtered projection data to the image domain. Using the notation of (1), the FDK algorithm is given by

(2) $$\begin{array}{c} \mathrm{FDK}\left(y,h\right)=W^{T}{\left(h*r(y)\right)}_{1D}, \end{array}$$

with $W^{T}$ the transpose of *W*. The operator $W^{T}$ is also known as the *backprojection operator*.

In [22,23,33], exponential binning is used to approximate filters, leading to $N_{e}\approx \log N$ coefficients to describe a filter. This approximation can be seen as a matrix $E\in R^{2N\times N_{e}}$ applied to a coefficient vector $h_{e}\in R^{N_{e}}$:

(3) $$\begin{array}{c} h\approx Eh_{e}. \end{array}$$

The implementation details of this filter approximation can be found in [23].

#### 3.1.3. Perceptron

In a similar manner as in [32], we define a *perceptron* or *node* $P:R^{l}\to R$ as a non-linear activation function σ:R→R applied to a weighted sum of the input $\eta \in R^{l}$ with the weights $\xi \in R^{l}$ and a bias b∈R:

(4) $$\begin{array}{c} P_{\xi ,b}\left(\eta \right)=\sigma \left(\eta \cdot \xi -b\right) \end{array}$$

In this paper, we will only consider the sigmoid function as an activation function, i.e., $\sigma \left(t\right)=1/\left(1+e^{-t}\right)$.

A multilayer perceptron is a network structure containing two types of layers with perceptrons, where each perceptron operates on the outputs of the previous layer. These layers are, in order, any number of *hidden layers*, and the *output layer*. Note that the number of hidden layers and number of hidden nodes $N_{h}$ in these layers can be chosen freely.

### 3.2. Reconstruction Algorithm & Network Design

We formulate the NN-FDK reconstruction algorithm in a similar fashion as the NN-FBP method in [33]. The NN-FDK reconstruction algorithm consists of $N_{h}$ individual FDK algorithms executed on the input data *y*, each using its own (exponentially binned) filter $h_{e}^{k}\in R^{N_{e}}$. It combines these $N_{h}$ volumes into a single reconstruction, using point-wise application of the activation function σ and an output perceptron with parameters $b_{o},b_{k}\in R$, and $\xi \in R^{N_{h}}$.

We use $\theta =(\xi ,b_{o},h_{e}^{k},b_{k})$ as short-hand for the full set of parameters of the NN-FDK reconstruction algorithm. The full algorithm (Algorithm 1) is then given by the following equation.

(5) $$\begin{array}{c} {\text{NN-FDK}}_{\theta }\left(y\right)=\sigma \left(\sum_{k=1}^{N_{h}}\xi_{k}\sigma \left(\mathrm{FDK}(y,Eh_{e}^{k}\right)-b_{k})-b_{o}\right) \end{array}$$

The FDK algorithm is a bilinear map in the input projection data and the used filter. Therefore, for fixed input projection data y and an expanded exponentially binned filter $Eh_{e}$, the FDK algorithm can be written as a linear map *F*_**y**_ applied to $Eh_{e}$. The product *F*_**y**_*E* can be considered as a matrix of size $N^{3}\times N_{e}$, and the *v*-th voxel of the output of the FDK algorithm is given by the inner product of **h**_*e*_ with ${\left(F_{y}E\right)}_{v:}$, the *v*-th row of the matrix *F*_**y**_*E*. This leads to the following:

(6) $$\begin{array}{cc} {\left({\text{NN-FDK}}_{\theta }\left(y\right)\right)}_{v} & =\sigma \left(\sum_{k=1}^{N_{h}}\xi_{k}\sigma {\left((F_{y}Eh_{e}^{k}\right)}_{v}-b_{k})-b_{o}\right), \end{array}$$

(7) $$\begin{array}{cc} \; & =\sigma \left(\sum_{k=1}^{N_{h}}\xi_{k}\sigma {\left((F_{y}E\right)}_{v:}h_{e}^{k}-b_{k})-b_{o}\right), \end{array}$$

(8) $$\begin{array}{cc} \; & =P_{\xi ,b_{o}}\left({\left[P_{h_{e}^{k},b_{k}}\left({\left(F_{y}E\right)}_{v:}\right)\right]}_{k}\right). \end{array}$$

Therefore, we define the two-layer perceptron network $N_{\theta }:R^{N_{e}}\to R$:

(9) $$\begin{array}{c} N_{\theta }\left(q\right)=P_{\xi ,b_{0}}\left({\left[P_{h_{e}^{k},b_{k}}\left(q\right)\right]}_{k}\right). \end{array}$$

This is our NN-FDK network, and as we derived above, it has the following relationship with the NN-FDK reconstruction algorithm:

(10) $$\begin{array}{c} N_{\theta }\left({\left(F_{y}E\right)}_{v:}\right)={\left({\text{NN-FDK}}_{\theta }\left(y\right)\right)}_{v}. \end{array}$$

**Figure 1:**
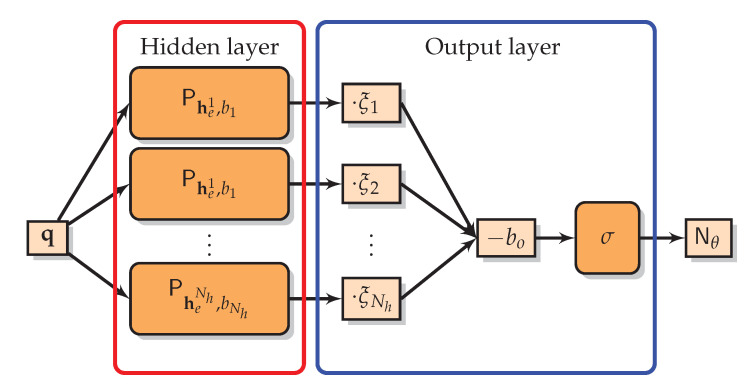
Schematic representation of the NN-FDK network, $N_{\theta }:R^{N_{e}}\to R$, with $N_{h}$ hidden nodes. Note that if we take $q={\left(F_{y}E\right)}_{v:}$ we get $q\cdot h_{e}^{k}={\left(\mathrm{FDK}\left(y,Eh_{e}^{k}\right)\right)}_{v}$ in the perceptrons of the hidden layer and the output of the network is equal to the *v*-th voxel of the NN-FDK reconstruction algorithm.

This relationship shows that we can *evaluate* the NN-FDK reconstruction algorithm efficiently on full input projection data at once, but also *train* the NN-FDK network efficiently with each *individual* voxel ${\left(x_{\mathrm{HQ}}\right)}_{v}$ in a high-quality reconstruction yielding a training pair with input ${\left(F_{y}E\right)}_{v:}$ and target ${\left(x_{\mathrm{HQ}}\right)}_{v}$. A schematic representation of the network is given in Figure 1.

Note that we arrive at the same network structure as found in [33] for FBP, using only the properties that the FDK algorithm is a bilinear map in the data and the filter, and that all operations can be applied point-wise. Using this reasoning, we can derive a similar network structure for any FBP-type method satisfying these conditions.

Even though we use the same network structure as [33], the way we compute inputs to the network is different. In [33], the input to the NN-FBP network is explicitly calculated by shifting and adding projection data for each reconstruction pixel. The FDK algorithm has additional weighting factors and lacks the shift-invariance property, which makes the approach presented in [33] not directly applicable. In the next section, we detail an alternative method to compute the input. The same approach could be applied to the NN-FBP method, similarly simplifying the network input computations.


**Algorithm 1** Neural Network Feldkamp–Davis–Kress (NN-FDK) reconstruction algorithm
1: Given a set of parameters, $\theta :=\left(\xi ,b_{o},h_{e}^{k},b_{k}\right)$.
2: Compute $H_{k}$ for all nodes *k* of the hidden layer:
3: **for**
$k=\{1,2,..,N_{h}\}$
**do**
4: $H_{k}\left(y\right)=\sigma \left(\mathrm{FDK}(y,Eh_{e}^{k}\right)-b_{k})$
5: **end for**6: Compute the output of the output layer: ${\text{NN-FDK}}_{\theta }\left(y\right)=\sigma \left(\sum_{k=1}^{N_{h}}\xi_{k}H_{k}\left(y\right)-b_{o}\right)$


### 3.3. Training Process

#### 3.3.1. Training and Validation Data

We will train our network using supervised learning, where we assume that we have $N_{\mathrm{TD}}$ and $N_{\mathrm{VD}}$ datasets available for training and validation, respectively. These datasets consist of low-quality tomographic input data and a high-quality reconstruction from which we randomly draw a total of $N_{T}$ training pairs and $N_{V}$ validation pairs. Note that we ensure that every drawn pair is unique and that an equal number of pairs is taken from each dataset. Moreover, to avoid selecting too many training pairs from the background, we only take training pairs from a region of interest (ROI) around the scanned object. This ROI is defined from the high-quality reconstruction as the voxels in the reconstructed object plus a buffer of roughly 0.2 N voxels around it.

Recall from the previous section that given low-quality tomographic data y and a high-quality reconstruction $x_{\mathrm{HQ}}$, the matrix *F*_**y**_*E* contains each input vector $Z={\left(F_{y}E\right)}_{v:}\in R^{N_{e}}$ corresponding to the target voxel $O={\left(x_{\mathrm{HQ}}\right)}_{v}$. However, due to memory constraints, *F*_**y**_*E* cannot be computed directly as a matrix product. Therefore, we observe that each column of *F*_**y**_*E* is an FDK reconstruction with a specific filter:

(11) $$\begin{array}{c} {\left(F_{y}E\right)}_{:j}=F_{y}Ee_{j}=\mathrm{FDK}\left(y,Ee_{j}\right), \end{array}$$

with $e_{j}\in R^{N_{e}}$ the unit vector with all entries equal to zero except for the *j*-th element.

#### 3.3.2. Learning Problem

The parameters of the NN-FDK network are learned by finding the set of parameters $\theta^{⋆}$ that minimize the *loss function* L on the training set. We minimize the $\ell^{2}$-distance between the network output and the target voxel for all training pairs in *T*:

(12) $$\begin{array}{cc} \theta^{⋆} & =\underset{\theta }{\mathrm{argmin}}L\left(\theta ,T\right)=\underset{\theta }{\mathrm{argmin}}\frac{1}{2}\sum_{j=1}^{N_{T}}{\left(O_{j}-N_{\theta }\left(Z_{j}\right)\right)}^{2}. \end{array}$$

To minimize the loss function, we use a quasi-Newton optimization scheme, the *Levenberg–Marquardt algorithm* (LMA) as proposed in [50,51]. This is a combination of gradient descent and the Gauss-Newton algorithm, improving the stability of Gauss-Newton while retaining its fast convergence and it is specifically designed to minimize a non-linear least squares problem such as (12). Note that the small number of parameters of the proposed network allows us to use such a method. Lastly, to avoid overfitting we check whether every update of the parameters also reduces the loss function on the validation set. We discuss the specifics of this algorithm in Appendix B.

### 3.4. Method Characteristics & Comparison

To conclude the method section, we compare the characteristics of the NN-FDK algorithm to those of several other methods. These methods are two 2D post-processing DNNs (U-net [31] and MSD-net [28]) applied in a slice-by-slice fashion, the SIRT^+^ algorithm [34] and the FDK algorithm. We focus our discussion on the goals formulated in Section 1 and show a summary of this comparison in Table 1. The reconstruction accuracy will be discussed in Section 5.

#### 3.4.1. Computational Efficiency

We approximate the reconstruction time by counting how many times it has to evaluate its most expensive computations. For simplicity, we assume that a backprojection takes approximately the same time as a forward projection, $T_{\mathrm{BP}}$.

- **FDK:** The FDK algorithm consist of one reweighting, filtering and backprojection step, i.e.,:
  (13) $$\begin{array}{c} T_{\mathrm{FDK}}\approx T_{\mathrm{BP}}. \end{array}$$
- **NN-FDK:** The NN-FDK algorithm performs one FDK reconstruction per hidden node $N_{h}$. Therefore, the reconstruction time becomes:
  (14) $$\begin{array}{c} T_{\text{NN-FDK}}\approx N_{h}T_{\mathrm{BP}}. \end{array}$$
- **SIRT^+^:** The SIRT^+^ method evaluates a forward and backprojection for each iteration. For $N_{\mathrm{iter}}$ iterations, the reconstruction time becomes:
  (15) $$\begin{array}{c} T_{{\mathrm{SIRT}}^{+}}\approx 2N_{\mathrm{iter}}T_{\mathrm{BP}}. \end{array}$$
- **DNN:** To evaluate a DNN on a full 3D volume, an FDK reconstruction is performed and a 2D network is applied per slice of the FDK reconstruction.
  (16) $$\begin{array}{c} T_{\mathrm{DNN}}^{3D}\approx T_{\mathrm{BP}}+NT_{\mathrm{DNN}}^{2D}, \end{array}$$
  with $T_{\mathrm{DNN}}^{2D}$ the time it takes to apply a 2D DNN and $T_{\mathrm{DNN}}^{3D}$ the time to do a full DNN reconstruction.

On a modern Nvidia GeForce GTX 1080Ti (11 GB) GPU and with N=1024 and $N_{a}=360$, we found in our experiments that $T_{\mathrm{BP}}\approx 10$ s and $T_{\mathrm{DNN}}^{2D}\approx 0.5$ s.

Comparing the reconstruction times, we see that NN-FDK is similar to FDK when the number of nodes $N_{h}$ is small, which is the case since we will take $N_{h}=4$ (see Section 4.3). For DNNs, the computational load of applying a 2D network leads to relatively high reconstruction times compared to the FDK algorithm. Lastly, we note that the number of iterations $N_{\mathrm{iter}}$ often lies between the 20 and 200, making SIRT^+^ several times slower than the (NN-)FDK algorithm.

#### 3.4.2. Number of Trainable Parameters

The number of trainable parameters is closely related to the amount of training data required to train a network [28]. From the definition of the NN-FDK network (5), we can compute the number of trainable parameters |θ|:

(17) $$\begin{array}{c} \left|\theta \right|=\left(N_{e}+2\right)N_{h}+1, \end{array}$$

with $N\gg N_{h},N_{e}>0$. Taking $N_{h}=4$ and N=1024 gives |θ|=61, which is several orders of magnitude lower than the typical numbers of parameters in a DNN (several tens of thousands to millions).

#### 3.4.3. Training Time

In the training step, a solution to the minimization problem (12) is computed. For the NN-FDK algorithm, this problem has $N_{T}$ samples and |θ| unknowns. In a similar fashion, we can formulate a least squares problem for training a DNN. Even assuming that we only take the same number of training samples to train the DNNs, this least squares problem is already orders of magnitude larger than that for NN-FDK due to the difference in the number of trainable parameters. Moreover, the LMA (the algorithm used to train NN-FDK) approaches quadratic convergence, which means it will need fewer iterations to converge than a first-order scheme such as ADAM [52], which is often used for training DNNs. Considering these two observations, we expect the training time of the NN-FDK algorithm to be lower than the training time of the DNNs.

## 4. Experimental Setup

We carried out a range of experiments to assess the performance of the NN-FDK algorithm with respect to the goals formulated in Section 1 compared to several alternative methods. In this section, we introduce the setup of these experiments. We describe the simulated data in Section 4.1 and the experimental data in Section 4.2. In Section 4.3, we discuss the specific network structure for the NN-FDK algorithm and the training parameters used. Finally, we give the quantitative measures we use to compare the reconstruction in Section 4.4.

### 4.1. Simulated Data

**Figure 2:**
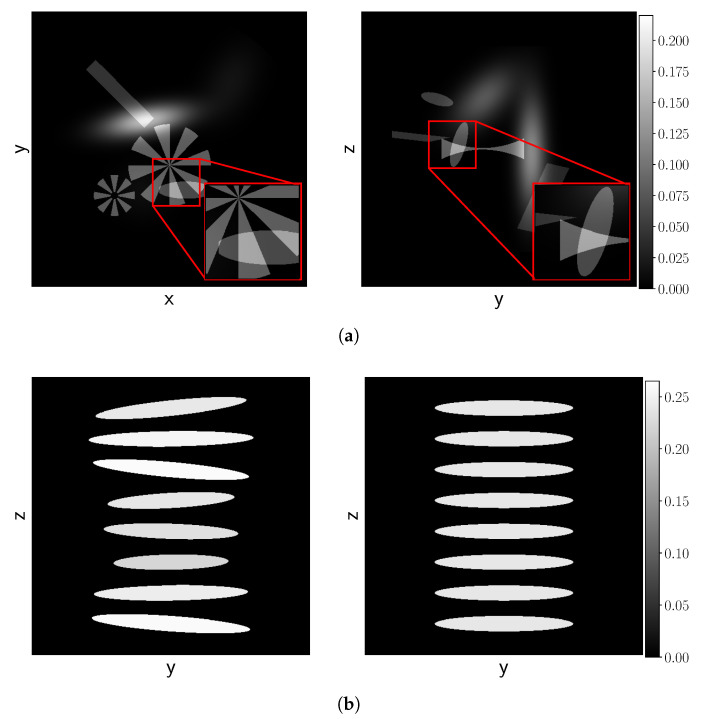
Examples simulated data. (**a**) Slices, (**Left**) z=0, (**Right**) x=0, of the Fourshape test phantom. This phantom is designed such that at least one of all objects can clearly be observed in the slices. (**b**) The x=0 slice for a Random Defrise phantom (**Left**) and the standard Defrise phantom without alternating intensities from [53] (**Right**).

We consider two types of phantom families for the simulated data experiments: the *Fourshape phantom family* and the *Random Defrise phantom family*. Examples are shown in Figure 2a,b, respectively. The Fourshape phantom family contains three random occurrences of each of four types of objects: an ellipse, a rectangle, a Gaussian blob and a Siemens star. For evaluation and visualization of the reconstructions, we fixed one realization that clearly shows at least one of all the four objects and we will refer to this phantom as the *Fourshape test phantom*. The Random Defrise phantom family is a slight adaptation of the phantom introduced in [53], which is a common phantom for assessing the influence of imaging artifacts due to the cone angle. Here, we vary the intensities, orientations and sizes of the disks making sure they do not overlap. Again, we define a test phantom for evaluation and visualization, which is in this case, the standard *Defrise phantom* without alternating intensities (right in Figure 2b). To simulate realistic settings, we scale the phantoms to fit inside a 10 cm cube, and use an attenuation coefficient of μ=0.22 cm^−1^, approximating that of various common plastics at 40 keV [54]. These phantoms are defined through geometric parameters, and can, therefore, be generated for any desired *N*. For our experiments, we will take N=1024. Details about how we generate the data are given in Appendix A.1.

To compute a high-quality reconstruction $x_{\mathrm{HQ}}$ that can be used as target for training (recall Section 3.3), we consider a simulated dataset with $N_{a}=1500$ projection angles, low noise ($I_{0}=2^{20}$ emitted photon count) and cone angle of 0.6 degrees and reconstruct this problem with the standard FDK algorithm using a Hann filter [9].

### 4.2. Experimental Data

For experimental data, we consider a set of CT scans that were recorded using the custom-built and highly flexible FleX-ray CT scanner, developed by XRE NV and located at CWI [55]. This scanner has a flat panel detector with 972×768 pixels and a physical size of 145.34×114.82 mm. This set of 42 scans was set up to create high-noise reconstruction problems and low-noise reconstruction problems with a low number of projection angles.

**Figure 3:**
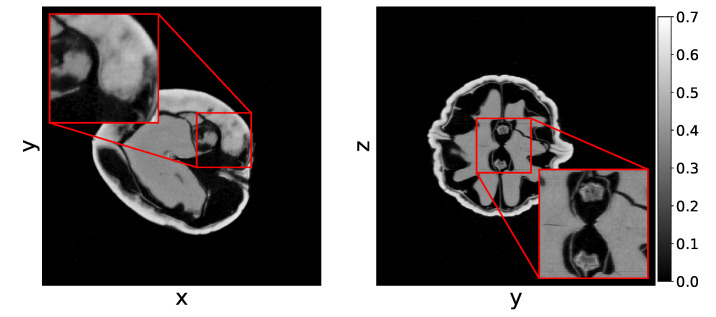
The z=0 (**Left**) and y=0 (**Right**) slice of the gold standard reconstruction of the high-dose dataset of the 21st walnut with full number of projection angles. The projection data are acquired using the FleX-ray scanner located at the CWI [55]. The horizontal line artifacts visible in the right figure are due to imperfections in the projection data and not due to the reconstruction process.

We acquired high-dose (low noise) and low-dose (high noise) scans of 21 walnuts. The datasets contain 500 equidistantly spaced projections over a full circle. The distance from the center of rotation to the detector was set to 376 mm and the distance from the source to the center of rotation was set to 463 mm. The scans were performed with a tube voltage of 70 kV. The high-dose scan was collected with a tube power of 45 W and an exposure time of 500 ms per projection. The low-dose scan was collected with a tube power of 20 W and an exposure time of 100 ms per projection. To create a low noise reconstruction problem with a low number of projection angles, we considered the high-dose scan but only took every 16-th projection angle. As high-quality reference reconstructions, we used SIRT${}^{+}$ reconstructions with 300 iterations (SIRT${}_{300}^{+}$) of the high-dose scans with all available projection angles ($N_{a}=500$). We will refer to these reconstructions as the *gold standard* reconstruction and we show such a reconstruction in Figure 3. These datasets are available at Zenodo [56].

### 4.3. Parameter Settings NN-FDK

In our initial experiments, we found that taking more FDK-perceptrons improved the accuracy of the networks, at the cost of increasing the training and reconstruction time. More specifically, the reconstruction time scales linearly with the number of perceptrons, whereas the reconstruction accuracy only shows marginal improvements for more than $N_{h}=\;4$ FDK-perceptrons, which is similar to the findings in [33]. Therefore, we fix the number of FDK-perceptrons at $N_{h}=\;4$, and denote the resulting network structure by NN-FDK${}_{4}$.

We found that, similar to the findings in [33], taking $N_{T}=10^{6}$ voxels for training and $N_{V}=10^{6}$ for validation is sufficient for training an NN-FDK network. As test data, we randomly generate 20 datasets for simulated data. For the experimental data, we use all datasets that were not used in the training process.

The network structures and training procedure used for the U-nets and MSD-nets are discussed in Appendix A.2.

### 4.4. Quantitative Measures

To quantify the accuracy of the reconstructions, we consider two measures, the test set error (TSE) and the structural similarity index (SSIM). These measures compare the reconstructed image $x_{r}$ to a high-quality reconstruction $x_{\mathrm{HQ}}$ on the ROI (as discussed in Section 3.3).

The TSE is the average loss (as defined in (12) in Section 3.3) of the test set, where the test set is all the voxels defined in the ROI of $x_{\mathrm{HQ}}$:

(18) $$\begin{array}{cc} \mathrm{TSE}(x_{r},x_{\mathrm{HQ}}) & =\frac{1}{N_{\mathrm{ROI}}}L\left(I_{\mathrm{ROI}}\left(x_{\mathrm{HQ}}\right),\theta \right), \end{array}$$

(19) $$\begin{array}{cc} \; & \;\;\;\;\;\;=\frac{1}{2N_{\mathrm{ROI}}}{\left\|I_{\mathrm{ROI}}\left(x_{\mathrm{HQ}}-x_{r}\right)\right\|}_{2}^{2}. \end{array}$$

with $I_{\mathrm{ROI}}:R^{N^{3}}\to R^{N^{3}}$ the masking function for the ROI and $N_{\mathrm{ROI}}$ the number of voxels in the ROI.

The SSIM [57] is implemented based on the scikit-image 0.13.1 [58] package, where all the constants are set to default and the filter is uniform, with a width of 19 pixels.

## 5. Results and Discussion

### 5.1. Scalability

#### 5.1.1. Memory Scaling

**Figure 4:**
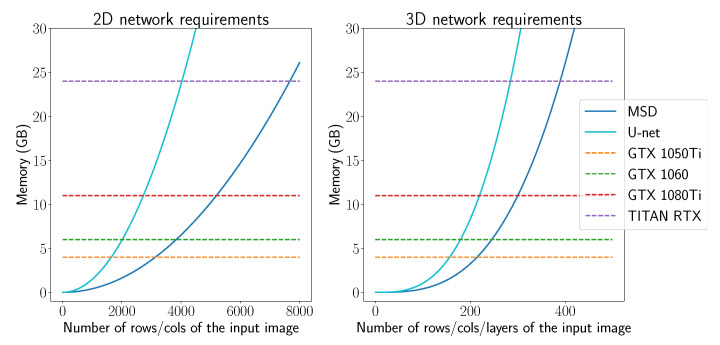
The required memory to store all intermediate images for applying a 2D and 3D U-net and MSD-net as a function of the input image size.

The required memory to store all intermediate images for a forward pass of a 2D or a 3D U-net and MSD-net as a function of the input image size is shown in Figure 4. Considering that CT imaging problems typically range from 256×256×256 up to 4096×4096×4096, we conclude from these figures that full 3D networks do not fit into GPU memory for higher resolutions and that even for 2D U-nets, not all resolutions fit into the GPU. As a forward pass of the NN-FDK algorithm requires only one additional reconstruction volume compared to the FDK algorithm, the memory requirements of the NN-FDK algorithm are roughly two-times the memory required by the FDK algorithm. (Technically, a forward pass of the NN-FDK algorithm can be done for every voxel separately; however, for the sake of comparison we assume a forward pass is for a full reconstruction volume.)

#### 5.1.2. Training Time

**Figure 5:**
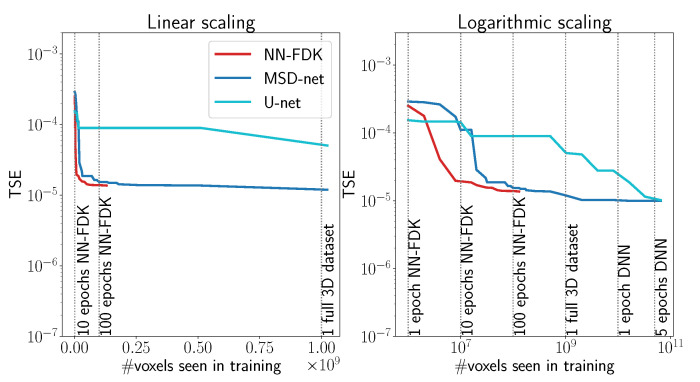
The test set error (TSE) as a function of the number of voxels the training process has seen. We report the lowest TSE until that point. The networks are trained on randomly generated Fourshape phantoms with size N=1024, $N_{a}=32$ projection angles and no noise. (**Left**) Linear scaling in the number of voxels ranging from 1 epoch for the NN-FDK ($10^{6}$ voxels), to one full 3D dataset ($10^{9}$ voxels). (**Right**) Logarithmic scaling in the number of voxels. Ranging from 1 epoch for the NN-FDK network ($10^{6}$ voxels) to 5 epochs for a DNN ($5\times 10^{10}$ voxels).

In Figure 5, we compare the training processes by plotting the progress of the network training (measured by the TSE) as a function of the number of voxels that the network has seen during training. We see that the NN-FDK has seen $1.1\times 10^{8}$ voxels when it converges to TSE $=1.4\times 10^{-5}$, whereas, MSD-net and U-net have seen $5.1\times 10^{8}$ voxels and $3.2\times 10^{9}$ voxels, respectively, at the point they first achieve a similar TSE. Important to note is that both U-net and MSD-net are not yet converged when they match the TSE of NN-FDK, and in general, the DNNs achieve lower TSEs than NN-FDK.

In Table 2, we show various timings and properties with respect to the training process. These timings are recorded using one Nvidia GeForce GTX 1080Ti with 11GB memory. We define a converged training process as 100 epochs without improvement on the validation set error and the number of epochs to converge as the epoch with the lowest validation set error during a converged training process. From these results, we see that the size of the training problem influences the time per epoch as an NN-FDK epoch is sub-second and the time per epoch for DNNs is in the range of hours.

In practice, we observed that after 2 days of training for the DNNs, any additional training only achieved marginal improvements. Therefore, in the following experiments, we train all DNNs for 2 days with one Nvidia GeForce GTX 1080Ti GPU, unless mentioned otherwise.

#### 5.1.3. Reconstruction Time

We measured the average reconstruction times and corresponding standard deviation over 120 reconstructions with resolution $N^{3}=1024^{3}$ and $N_{a}=360$ projection angles. These reconstructions are computed using one Nvidia GeForce GTX 1080Ti with 11 GB memory. The results are shown in Table 3. The subscript 200 for SIRT${}^{+}$ denotes the number of iterations that were used for the reconstruction. We define the reconstruction time as the time it takes to compute the full 3D volume. This means for U-net and MSD-net, an FDK reconstruction needs to be computed and the network needs to be applied N=1024 times to a 2D slice. Although every application can be done within a second (U-net ≈0.3 s, MSD-net ≈0.7 s) this leads to long reconstruction times.

### 5.2. Reconstruction Accuracy for Simulated Data

For evaluating the reconstruction accuracy using simulated data, we consider 16 cases: 6 different noise levels, 5 different numbers of projection angles and 5 different cone angles. For each case, an NN-FDK, MSD-net and U-net network was trained. For the training process of NN-FDK, we used $N_{T}=10^{6}$ training voxels and $N_{V}=10^{6}$ validation voxels from $N_{\mathrm{TD}}=10$ and $N_{\mathrm{VD}}=5$ datasets, respectively. For U-net and MSD-net, we took the same datasets for training and validation (10 for training and 5 for validation), and used all voxels in these datasets for the training process. The NN-FDK networks were trained till convergence and the DNNs were trained for 48 h. Note that in a few cases, we had to retrain the DNNs because of inconsistent results (i.e., cases with more information achieving a lower reconstruction accuracy), possibly because they got stuck in local minima of the loss function.

**Figure 6:**
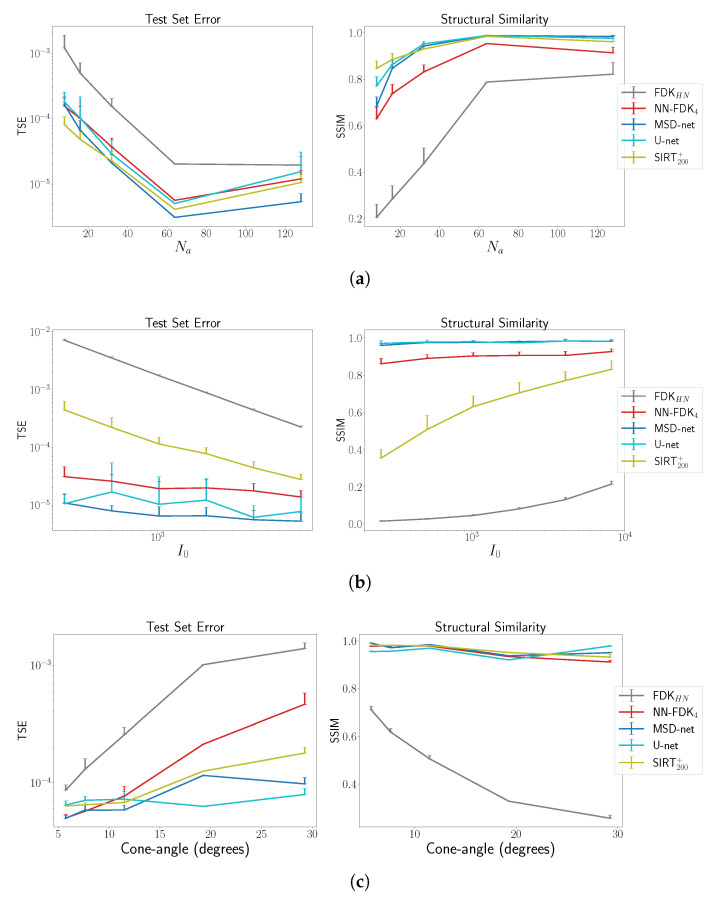
The average and standard deviation of the TSE andstructural similarity index (SSIM). These results are discussed in Section 5.2. For each number of projection angles, the noise level, cone angle and training scenario of one specific network are trained and used to evaluate the 20 reconstruction problems. The NN-FDK reconstruction time is 4-10 times lower than U-net, MSD-net and approximately 40 times lower than SIRT${}_{200}^{+}$. (**a**) The average and standard deviation of the TSE and SSIM as a function of number of projection angles $N_{a}$ computed over 20 randomly generated phantoms Fourshape family. (**b**) The average and standard deviation of the TSE and SSIM as a function of the emitted photon count $I_{0}$ computed over 20 randomly generated phantoms of the Fourshape family. (**c**) The average and standard deviation of the average TSE and SSIM as a function of the cone angle computed over 20 randomly generated phantoms of the Defrise family.

**Figure 7:**
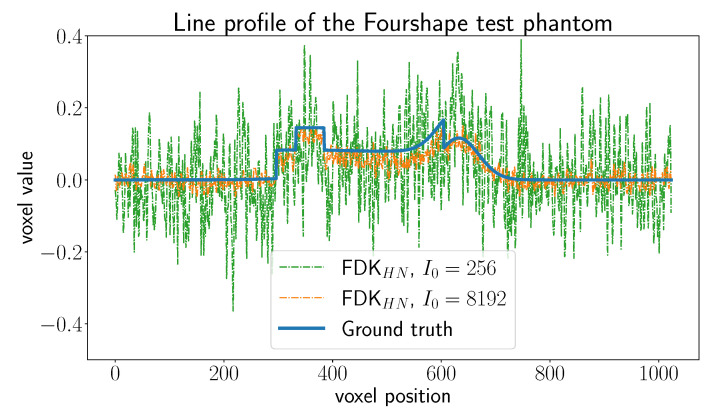
Line profile through the center of the z=0 slice of the Fourshape test phantom. We show the ground truth profile, the profile of the FDK reconstruction with lowest emitted photon count $I_{0}=256$, and the profile of the FDK reconstruction with the highest emitted photon count $I_{0}=8196$.

**Figure 8:**
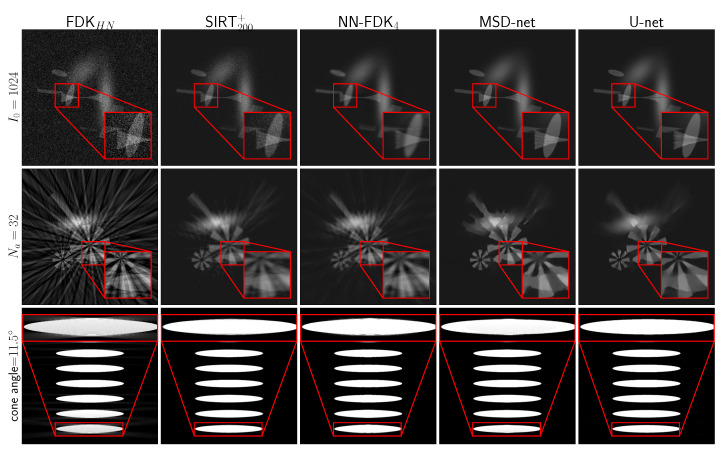
Two-dimensional slices of the reconstructions for the considered reconstruction methods. (Top) Slice x=0 of the Fourshape test phantom reconstruction problem with $N_{a}=360$ projection angles and $I_{0}=1024$ emitted photon count. (Middle) Slice z=0 of the Fourshape test phantom reconstruction problem with $N_{a}=32$ projection angles. (Bottom) Slice x=0 of the Defrise reconstruction problem with $N_{a}=360$ projection angles and a cone angle of 11.5 degrees.

In Figure 6, we show the average and standard deviation of the TSE and the SSIM for the considered cases. The subscript HN for the FDK algorithm indicates that the *Hann* filter was used to compute the reconstruction. We observe that U-net and MSD-net achieve the most accurate results and that NN-FDK and SIRT${}^{+}$ closely follow. The FDK algorithm is lowest in all categories. Between NN-FDK and SIRT${}^{+}$, we see that NN-FDK performs best for the noisy reconstruction problems and SIRT${}^{+}$ achieves better results for the reconstruction problems without noise. We visualize the noise for the lowest and highest $I_{0}$ in Figure 7 by showing a line profile through the center of the z=0 slice. Here, we see that for the noisiest problems, the amplitude of the noise can be as high as the maximum value of the phantom. In Figure 8, we show 2D slices of reconstructions of the test phantoms for the three types of reconstruction problems. In all cases, we still observe reconstruction artifacts, but comparing these to the baseline FDK reconstructions, the majority is removed or suppressed.

### 5.3. Reconstruction Accuracy for Experimental Data

In this section, we use the datasets discussed in Section 4.2 to assess the reconstruction accuracy of experimental data. In a similar fashion as for the simulated data, we trained a network for the low-dose reconstruction problem and a network for the high-dose reconstruction problem with $N_{a}=32$ projection angles with the notable exception that U-net and MSD-net were trained till convergence. The results are presented in Table 4.

**Figure 9:**
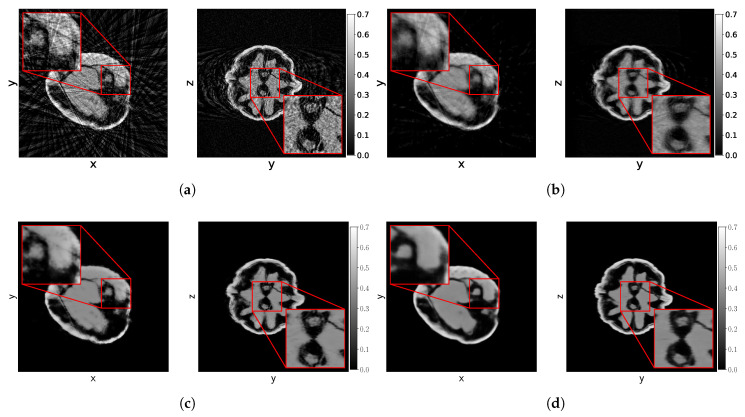
Slices z=0 and x=0 of several reconstruction methods of the high-dose dataset of the 21 st walnut with 32 projection angles. (**a**) FDK${}_{\mathrm{HN}}$. (**b**) SIRT${}_{200}^{+}$ reconstruction. (**c**) NN-FDK${}_{4}$ reconstruction. (**d**) MSD-net reconstruction.

Comparing the results to the simulated data experiments, we see that SIRT${}^{+}$ performs worse on the experimental data, even with the additional regularization of early stopping. This is most likely due to the high-dose datasets still containing noise, whereas this was completely absent in the simulated data experiments. These differences are illustrated in Figure 9, where 2D slices of the reconstructions for the high-dose reconstruction problem with $N_{a}=32$ projection angles are shown.

### 5.4. Segmentation Experiment for Experimental Data

To assess the performance of the different reconstruction approaches in a segmentation task, we focus here on the segmentation of the shell and kernel of walnuts, based on our experimental CT data. The review [59] provides an overview of segmentation problems in walnut imaging, and their relevance. For segmenting the 3D volume after the reconstruction, we used a deterministic segmentation algorithm that combines thresholding, the watershed algorithm and prior knowledge of the scanned objects. Details of this method are discussed in Appendix A.4. For the reference segmentation, we apply this algorithm to the gold standard reconstruction.

For determining the accuracy of the segmentation of an object—i.e., shell, empty space and kernel of the walnut—we consider three metrics: volume error, mislabeled voxels and the Dice coefficient [60]. We define a segmentation *S* as a reconstruction volume with value 1 if the voxel is in the object (shell, kernel or empty space) and 0 if outside the object. Furthermore, we define the norm of a segmentation as the sum: $\left|S\right|=\sum_{i}^{N^{3}}{\left(S\right)}_{i}$. Using this notation, we can compute the measures in the following manner:

(20) $$\begin{array}{ccccc} V_{\mathrm{err}} & =\frac{|S_{\mathrm{rec}}\left|-\right|S_{\mathrm{GS}}|}{|S_{\mathrm{GS}}|}, & {\mathrm{ML}}_{\mathrm{err}} & =\frac{|S_{\mathrm{rec}}-S_{\mathrm{GS}}|}{|S_{\mathrm{GS}}|}, & \mathrm{DC}=\frac{2|S_{\mathrm{rec}}\cap S_{\mathrm{GS}}|}{|S_{\mathrm{rec}}\left|+\right|S_{\mathrm{GS}}|}, \end{array}$$

with GS denoting the gold standard reconstruction.

In Table 5, we show the results for computing these metrics on the 6 walnuts not considered in the training process. We observe that MSD-net performs best in segmenting the shell and U-net performs best at segmenting the empty space and kernel and NN-FDK is close to both DNNs and in some cases even better than MSD-net for segmenting the empty space and kernel. Comparing NN-FDK to standard FDK, we observe a significant improvement.

### 5.5. Data Requirements

To test the influence of the amount of training data on the reconstruction quality, we performed an experiment with three different training scenarios:

- **Scenario 1**. One dataset available. Here, we take the training and validation data from the same dataset.
- **Scenario 2**. Two datasets available. Here, we take the training and validation data from the separate datasets.
- **Scenario 3**. Fifteen datasets available. Again, the training and validation data are picked from separate datasets, but now the training and validation pairs come from several datasets, specifically 10 training datasets ($N_{\mathrm{TD}}=10$) and 5 validation datasets ($N_{\mathrm{VD}}=5$). This is the scenario used in the previous experiments.

We fix the number of voxels used for training and validation at $N_{T}=10^{6}$ and $N_{V}=10^{6}$ for all scenarios. For comparison, we trained a U-net and a MSD-net network with the same training scenarios, with the exception that all voxels from the datasets are used. For training scenario 1, the slices are divided into a training and a validation set. More specifically, every fourth slice is used for validation.

We performed this experiment for two simulated data problems, a high noise level (emitted photon count $I_{0}=256$) and a large cone angle (29.3 degrees), and the two experimental data problems. For the sake of brevity, we show only the results for the high-noise simulated data reconstruction problem (Table 6) and the high-noise experimental data reconstruction problem (Table 7). The results for the other reconstruction problems are given in Appendix C. Comparing quantitative measures between the different scenarios, we see that the reconstruction accuracy improves as more data is used for the simulated data experiment, but remains about the same for the experimental data experiment. This can be explained by the variation in the objects used in the reconstruction problems. Recall that the Fourshape phantom family has a large variety in its phantoms, i.e., three instances of four randomly generated objects, and the variety within the walnut datasets is small, i.e., similar shapes, sizes and structures. This indicates that if objects are similar, one training dataset may already be sufficient to train networks that achieve a high reconstruction accuracy.

Note that although the training scenarios for NN-FDK and the DNNs use the same number of datasets, the number of voxels considered for training the NN-FDK network is constant over all three scenarios and is several orders of magnitude lower than the number of voxels considered for training the DNNs. This opens up future possibilities for reducing the training data requirements to only need a high-quality reconstruction of a certain region of interest.

## 6. Summary and Conclusions

We have proposed the Neural Network FDK (NN-FDK) algorithm, a reconstruction algorithm for the circular cone-beam (CCB) Computed Tomography (CT) geometry with a machine learning component. The machine learning component of the algorithm is designed to learn a set of FDK filters and to combine the FDK reconstructions done with these filters. This leads to a computationally efficient reconstruction algorithm, since one only needs to compute and combine the FDK reconstructions for this learned set of filters. Due to parametrization of the learned filters, the NN-FDK network has a low number of trainable parameters (<100) and can be trained efficiently with the Levenberg–Marquardt algorithm with approximate quadratic convergence rate.

We compared the NN-FDK algorithm to SIRT with a nonnegativity constraint (SIRT${}^{+}$), the standard FDK algorithm and two deep neural networks (DNNs), namely a 2D U-net and a 2D MSD-net applied in a slice-by-slice fashion to a 3D volume. We have shown that the NN-FDK algorithm has the lowest reconstruction time after the standard FDK algorithm. We have also shown that the NN-FDK algorithm achieves a reconstruction accuracy that is similar to that of SIRT${}^{+}$ for simulated data and a higher accuracy than that of SIRT${}^{+}$ for experimental data. The DNNs achieved the highest reconstruction accuracy, but training those networks took between 2 days (1 training and validation dataset) and 2 weeks (15 training and validation datasets), whereas all the NN-FDK networks were trained within 1 minute.

To conclude, the NN-FDK algorithm is a computationally efficient reconstruction algorithm that can reconstruct CCB CT reconstruction problems with high-noise, low projection angles or large cone angles accurately. The training process is efficient and requires a low amount of training data, making it suitable for application to a broad spectrum of large scale (up to 4096×4096×4096) reconstruction problems. Specifically, the NN-FDK algorithm can be used to improve image quality in high-throughput CT scanning settings, where FDK is currently used to keep pace with the acquisition speed using readily available computational resources.

## Figures and Tables

**Table 1:** Comparison of reconstruction methods with respect to the goals formulated in Section 1. We consider a DNN to be 2D deep convolutional neural network (U-net and MSD-net) applied in slice-by-slice fashion to a standard FDK reconstruction. Reconstruction accuracy is defined as the accuracy of a method when reconstructing low-quality data, e.g., data with high noise or a low number of projection angles.

	Reconstruction		Training	
**Method**	**Time**	**Accuracy**	**Data**	**Time**
NN-FDK	++	?	++	+++
DNN	±	+++	±	- - -
FDK	+++	- -		
SIRT${}^{+}$	- -	+		

Method comparison: Goals.

**Table 2:** Timings and properties of the considered training processes. We define a converged training process as 100 epochs without improvement on the validation set error. The number of epochs to converge is, therefore, the epochs computed of such a process minus 100. The training was performed using one Nvidia GeForce GTX 1080Ti GPU (11 GB).

	NN-FDK${}_{4}$	MSD-net	U-net
Voxels seen in one epoch	$1\times 10^{6}$	$1.1\times 10^{10}$	$1.1\times 10^{10}$
Time per epoch	0.1336 s	0.95 h	2.36 h
Time to converge	28 s	±10 days	±14 days
Epochs to converge	110	128	42
Epochs in 2 days	-	45	18

Training process.

**Table 3:** Average and standard deviation of the reconstruction times (in seconds) computed over 120 reconstruction problems with N=1024 and $N_{a}=360$ projection angles. These reconstructions are computed using one Nvidia GeForce GTX 1080Ti GPU (11 GB).

FDK	SIRT${}_{200}^{+}$	NN-FDK${}_{4}$	U-net	MSD-net
28 ± 8	3225 ± 916	76 ± 8	382 ± 69	809 ± 86

Reconstruction times.

**Table 4:** Average and standard deviation of the quantitative measures computed over 6 walnut datasets. The high-dose low projection angle reconstruction problem has $N_{a}=32$ projection angles, the low-dose reconstruction problem has $N_{a}=500$ projection angles. For the high-dose data, we used 200 iterations of SIRT, and for the low-dose data, we used 20 iterations of SIRT. The NN-FDK reconstruction time is 4-10 times lower than U-net, MSD-net and SIRT${}_{20}^{+}$, and approximately 40 times lower than SIRT${}_{200}^{+}$.

	High-Dose, Low Number of Projection Angles		Low-Dose	
**Method**	**TSE**	**SSIM**	**TSE**	**SSIM**
FDK${}_{\mathrm{HN}}$	5.54 ± 3.43 $\times \;10^{-3}$	0.224 ± 0.076	1.40 ± 0.05 $\times \;10^{-3}$	0.334 ± 0.104
SIRT${}_{200/20}^{+}$	9.94 ± 0.15 $\times \;10^{-4}$	0.603 ± 0.087	1.92 ± 0.08 $\times \;10^{-3}$	0.584 ± 0.083
NN-FDK${}_{4}$	8.03 ± 1.39 $\times \;10^{-4}$	0.946 ± 0.010	1.14 ± 0.23 $\times \;10^{-4}$	0.965 ± 0.012
U-net	4.10 ± 1.06 $\times \;10^{-4}$	0.964 ± 0.009	1.02 ± 0.45 $\times \;10^{-4}$	0.980 ± 0.006
MSD-net	4.23 ± 0.97 $\times \;10^{-4}$	0.964 ± 0.009	7.82 ± 2.86 $\times \;10^{-5}$	0.980 ± 0.007

Experimental data.

**Table 5:** The average and standard deviation of the three metrics computed over the 6 low-dose walnut datasets with $N_{a}=500$ projection angles. The metrics are computed using (20). The NN-FDK reconstruction time is 4-10 times lower than U-net, MSD-net and approximately 40 times lower than SIRT${}_{200}^{+}$.

Method	Shell	Empty Space	Kernel
Volume errors			
FDK${}_{\mathrm{HN}}$	0.127 ± 0.078	0.146 ± 0.091	0.128 ± 0.092
SIRT${}_{200}^{+}$	0.082 ± 0.047	0.104 ± 0.078	0.050 ± 0.074
NN-FDK${}_{4}$	0.068 ± 0.035	0.045 ± 0.035	0.029 ± 0.032
U-net	0.055 ± 0.019	0.029 ± 0.017	0.012 ± 0.016
MSD-net	0.028 ± 0.010	0.059 ± 0.075	0.035 ± 0.050
Mislabeled voxels			
FDK${}_{\mathrm{HN}}$	0.168 ± 0.087	0.190 ± 0.98	0.144 ± 0.081
SIRT${}_{200}^{+}$	0.133 ± 0.026	0.182 ± 0.118	0.101 ± 0.048
NN-FDK${}_{4}$	0.103 ± 0.026	0.087 ± 0.023	0.072 ± 0.018
U-net	0.092 ± 0.028	0.073 ± 0.024	0.059 ± 0.019
MSD-net	0.086 ± 0.038	0.116 ± 0.094	0.061 ± 0.039
Dice coefficient			
FDK${}_{\mathrm{HN}}$	0.922 ± 0.036	0.895 ± 0.061	0.934 ± 0.033
SIRT${}_{200}^{+}$	0.934 ± 0.016	0.908 ± 0.061	0.947 ± 0.028
NN-FDK${}_{4}$	0.951 ± 0.012	0.955 ± 0.013	0.964 ± 0.008
U-net	0.955 ± 0.013	0.963 ± 0.012	0.971 ± 0.010
MSD-net	0.957 ± 0.018	0.939 ± 0.055	0.971 ± 0.018

Segmentation errors.

**Table 6:** Average and standard deviation of the quantitative measures computed over 20 Fourshape phantoms for varying training scenarios. The  reconstruction problems have an emitted photon count of $I_{0}=256$ and $N_{a}=360$ projection angles. The NN-FDK reconstruction time is 4-10 times lower than U-net and MSD-net.

TSE			
**Method**	**1 Dataset**	**2 Datasets**	**15 Datasets**
NN-FDK${}_{4}$	4.97 ± 4.68 $\times \;10^{-5}$	4.19 ± 3.60 $\times \;10^{-5}$	2.51 ± 1.14 $\times \;10^{-5}$
U-net	1.06 ± 1.36 $\times \;10^{-5}$	2.45 ± 2.87 $\times \;10^{-5}$	8.06 ± 3.63 $\times \;10^{-6}$
MSD-net	1.12 ± 0.41 $\times \;10^{-5}$	1.12 ± 0.40 $\times \;10^{-5}$	7.94 ± 3.16 $\times \;10^{-6}$
**SSIM**			
NN-FDK${}_{4}$	0.831 ± 0.065	0.844 ± 0.065	0.884 ± 0.030
U-net	0.884 ± 0.075	0.932 ± 0.050	0.979 ± 0.009
MSD-net	0.961 ± 0.013	0.962 ± 0.013	0.974 ± 0.008

Simulated data, high noise.

**Table 7:** Average and standard deviation of the quantitative measures computed over 6 walnuts for various training scenarios. The datasets are low-dose and have $N_{a}=500$ projection angles. The NN-FDK reconstruction time is 4-10 times lower than U-net and MSD-net.

TSE			
**Method**	**1 Dataset**	**2 Datasets**	**15 Datasets**
NN-FDK${}_{4}$	1.16 ± 0.25 $\times \;10^{-4}$	1.23 ± 0.25 $\times \;10^{-4}$	1.14 ± 0.23 $\times \;10^{-4}$
U-net	1.27 ± 0.38 $\times \;10^{-4}$	1.23 ± 0.35 $\times \;10^{-4}$	1.02 ± 0.45 $\times \;10^{-4}$
MSD-net	1.28 ± 0.41 $\times \;10^{-4}$	1.16 ± 0.35 $\times \;10^{-4}$	7.82 ± 2.86 $\times \;10^{-5}$
**SSIM**			
NN-FDK${}_{4}$	0.973 ± 0.009	0.968 ± 0.011	0.965 ± 0.012
U-net	0.979 ± 0.008	0.978 ± 0.008	0.980 ± 0.006
MSD-net	0.979 ± 0.008	0.979 ± 0.008	0.980 ± 0.007

Experimental data, low-dose.

## Abbreviations

The following abbreviations are used in this manuscript:

CCB	Circular cone-beam
CT	Computed Tomography
FDK	Feldkamp–Davis–Kress
NN-FDK	Neural Network Feldkamp–Davis–Kress
FBP	Filtered backprojection
DNN	Deep neural network
SIRT	Simultaneous Iterative Reconstruction Technique
MSD-net	Mixed-scale dense network
U-net	U-network
LMA	Levenberg–Marquardt algorithm

## Appendix A. Implementation

### Appendix A.1. Data Generation

For our simulated data experiments, we take N=1024, which means that reconstructions and reference images are defined on a $1024^{3}$ equidistant voxel grid, and the projection data on a $1024^{2}$ equidistant detector grid per projection angle. However, to avoid using the same operator for reconstructions as for the data generation, we generate the input data at a higher resolution. More specifically, we generate a phantom at N=1536, forward project this phantom to the data space with size $N_{a}\times 1536^{2}$ and apply a bilinear interpolation per projection angle to arrive at a $1024^{2}$ detector grid, resulting in input data with the desired resolution $N_{a}\times 1024^{2}$. We set the source radius to 10 times the physical size of the phantom, resulting in a cone angle of 5.7 degrees. To generate noise, we compute a noise-free photon count *I* from clean projection data $y_{c}$ and use that to generate a Poisson distributed photon count from which we compute **y**:

(A1) $$\begin{array}{cccccc} I & =I_{0}e^{-y_{c}}, & I_{noise}∼\mathrm{Pois}\left(I\right), & \; & y & =-\log(\frac{I_{noise}}{I_{0}}), \end{array}$$

with $I_{0}$ the emitted photon count. Higher $I_{0}$ implies a higher dose and, therefore, less noise in the data.

### Appendix A.2. Deep Neural Networks

**Application strategy:** We train 2D DNNs to remove artifacts from 2D slices of an FDK reconstruction. We train one network that handles all slices in the reconstructions.

**Training DNNs:** We train the DNNs with ADAM [52] and stop training after 48 h of training on an Nvidia GeForce GTX 1080Ti GPU, the network with the lowest validation set error during this training process will be used for the reconstructions.

**U-net and MSD-net structures:** For U-net, we will take four up and down layers with 3 × 3 convolutions, 2 × 2 max-pooling and 2 × 2 up-convolutions as presented in [31]. For the MSD-nets, we take 100 layers with one input and one output layer and the dilations, as suggested in [28].

### Appendix A.3. Code-Base

We implemented the NN-FDK framework using Python 3.6.5 and Numpy 1.14.5 [61]. For the parameter learning, we used the Levenberg–Marquardt algorithm implementation from [33]. The reconstruction algorithm is implemented using ODL [62], the ASTRA-toolbox [63], PyFFTW [64] and the exponential binning framework for filters from [23]. For performance reasons, the simulated phantoms are generated through C++ using Cython [65].

For the evaluation of U-nets, we took the PyTorch [66] implementation used in [67]. The MSD-nets are implemented using the package published with [26].

All the code related to this paper can be found on Github [68].

### Appendix A.4. Segmentation Algorithm

This algorithm consists of several steps:

1. Apply a Gaussian filter to the reconstruction.
2. Compute a histogram of the filtered reconstruction and determine the peaks relating to the background, kernel and shell.
3. Determine the shell and kernel segmentations using a threshold based on the found peaks.
4. Apply the watershed algorithm on the shell segmentation. This gives the total volume inside the walnut.
5. Remove the kernel from the total volume inside the walnut to attain the empty space segmentation.

Further details about this implementation can be found on our Github [68].

## Appendix B. Levenberg–Marquardt Algorithm

Given the learning problem (12), the update rule for the Levenberg–Marquardt algorithm (LMA) ([50,51]) is given by:

(A2) $$\begin{array}{c} \theta^{i+1}=\theta^{i}+t^{i}, \end{array}$$

with $t^{i}$ the update vector. This is computed by solving the following equation for $t^{i}$

(A3) $$\begin{array}{c} \left(J_{i}^{T}J_{i}+\lambda_{i}I\right)t^{i}=-\frac{\partial L}{\partial \theta }\left(\theta^{i},T\right)=-J_{i}^{T}\sum_{j=1}^{N_{T}}\left(O_{j}-N_{\theta }\left(Z_{j}\right)\right) \end{array}$$

where $\lambda_{i}>0$ is the step parameter and $J_{i}$ the m×n Jacobian matrix of $N_{\theta^{i}}\left(Z\right)$ with respect to $\theta^{i}$, with Z the vector containing all inputs from the training set *T*. We can solve (A3) using a Cholesky decomposition. ($J_{i}^{T}J_{i}$ is positive semi-definite and $\lambda_{i}>0$; therefore, the left-hand side of (A3) is positive definite.)

To ensure convergence, only updates that improve the training error are accepted, i.e., if the following is true:

(A4) $$\begin{array}{c} L\left(\theta^{i},T\right)>L\left(\theta^{i}+t^{i},T\right), \end{array}$$

If this is not the case, we change the step parameter $\lambda_{i}$ to $a\lambda_{i}$ with a>1 and compute a new update vector $t^{i}$. When an update is accepted, we change the step parameter to $\lambda_{i+1}=\lambda_{i}/a$.

We use two stopping criteria for the LMA. Firstly, we stop if we cannot find a suitable $\theta^{i+1}$, using several indicators for this:

- The norm of the gradient $\frac{\partial L}{\partial \theta }(\theta^{i})$ is too small
- The step size $\lambda_{i}$ is too big
- After $N_{\mathrm{up}}$ rejected updates.

The second stopping criterion checks whether the parameters $\theta^{i}$ improve the validation set error. More specifically, we terminate the LMA when the validation set error has not improved for $N_{\mathrm{val}}$ iterations.

In Algorithm A1, the LMA is summarized. The random initialization is done with the Nguyen–Widrow initialization method [69]. For our experiments, we take $N_{\mathrm{up}}=100$, $\lambda_{0}=10^{5}$, a=10 and $N_{\mathrm{val}}=100$.

**Algorithm A1** Levenberg–Marquardt algorithm
1: Compute random initialization $\theta^{0}$ using [69]
2: **repeat**
3: Compute $t^{i}$ until we accept an update $\theta^{i+1}$.
4: **until** $N_{\mathrm{up}}$ updates were rejected **or** $L(\theta^{i},V)$ did not improve $N_{\mathrm{val}}$ times **or** $\left\|\frac{\partial L}{\partial \theta }(\theta^{i+1})\right\|$ is too small **or** $\lambda_{i+1}$ is too big.
5: Set $\theta^{⋆}$ equal to the $\theta^{i}$ with the lowest validation error.

## Appendix C. Results Data Requirement Experiment

Results for simulated data with a large cone angle are shown in Table A1. Results for experimental data with high-dose adn 32 projection angles are shown in Table A2.

**Table A1:** Average and standard deviation of the quantitative measures computed over 20 different Defrise phantoms for various training scenarios. The reconstruction problems have a cone angle of 29.2 degrees and $N_{a}=360$ projection angles.

TSE			
**Method**	**1 Dataset**	**2 Datasets**	**15 Datasets**
NN-FDK${}_{4}$	6.47 ± 1.19 $\times \;10^{-4}$	4.70 ± 1.16$\times \;10^{-4}$	4.82 ± 1.13 $\times \;10^{-4}$
U-net	1.04 ± 0.27 $\times \;10^{-4}$	1.02 ± 0.17 $\times \;10^{-4}$	8.23 ± 0.85 $\times \;10^{-5}$
MSD-net	2.44 ± 1.43 $\times \;10^{-4}$	1.53 ± 0.17 $\times \;10^{-4}$4	6.52 ± 0.43 $\times \;10^{-5}$
**SSIM**			
NN-FDK${}_{4}$	0.825 ± 0.018	0.904 ± 0.011	0.910 ± 0.007
U-net	0.974 ± 0.015	0.971 ± 0.021	0.973 ± 0.010
MSD-net	0.954 ± 0.006	0.937 ± 0.004	0.966 ± 0.002

Simulated data, large cone angle.

**Table A2:** Average and standard deviation of the quantitative measures computed over the 6 datasets for various training scenarios. These are the high-dose datasets from [56] with $N_{a}=32$ projection angles. The best results are highlighted.

TSE			
**Method**	**1 Dataset**	**2 Datasets**	**15 Datasets**
NN-FDK${}_{4}$	8.14 ± 1.45 $\times \;10^{-4}$	8.68 ± 1.43 $\times \;10^{-4}$	8.03 ± 1.39 $\times \;10^{-4}$
U-net	7.56 ± 1.52 $\times \;10^{-4}$	6.85 ± 1.56 $\times \;10^{-4}$	**4.10 ± 1.06 $\times 10^{-4}$**
MSD-net	7.82 ± 0.41 $\times \;10^{-4}$	6.51 ± 0.35 $\times \;10^{-4}$	4.23 ± 0.97 $\times \;10^{-4}$
**SSIM**			
NN-FDK${}_{4}$	0.950 ± 0.010	0.948 ± 0.010	0.946 ± 0.011
U-net	0.955 ± 0.011	0.930 ± 0.023	**0.964 ± 0.009**
MSD-net	0.955 ± 0.010	0.947 ± 0.014	**0.964 ± 0.009**

Experimental data, high-dose, 32 projection angles.
